# Polymorphic Regulation of Outer Membrane Lipid A Composition

**DOI:** 10.1128/mBio.01903-16

**Published:** 2016-11-08

**Authors:** Russell E. Bishop

**Affiliations:** Department of Biochemistry and Biomedical Sciences and the Michael G. DeGroote Institute for Infectious Disease Research, McMaster University, Hamilton, Ontario, Canada

## Abstract

The importance of the polymorphic-phase behavior of lipid A structural variations in determining their endotoxic activities has been recognized previously, but any potential role for lipid A polymorphism in controlling outer membrane structure and function has been largely ignored until now. In a recent article in *mBio* [7(5):e01532-16, https://doi.org/10.1128/mBio.01532-16], Katherine E. Bonnington and Meta J. Kuehn of Duke University’s Department of Biochemistry make a compelling case for considering how the molecular shapes of the various lipid A structural subtypes found in the outer membrane contribute to the process of outer membrane vesicle (OMV) formation.

## COMMENTARY

**FIG 1:**
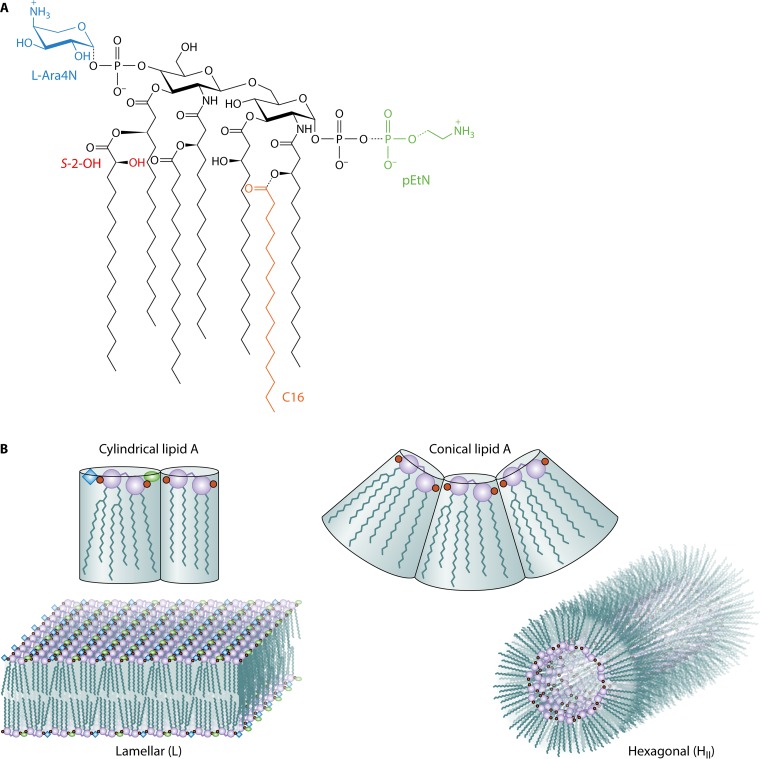
Structure of lipid A and the polymorphic regulation outer membrane lipid composition. (A) Lipid A in *S.* Typhimurium is an acylated and phosphorylated disaccharide of glucosamine linked by a β-1′,6-glycosidic bond. Unmodified lipid A exhibits four primary *R*-3-hydroxymyristate chains, where the two distal chains are esterified with secondary acyloxyacyl groups. *S*-2-OH can be incorporated into a myristate chain, and a palmitate chain (C_16_) can be incorporated as a regulated modification. Modification of the lipid A phosphate groups with phosphoethanolamine (pEtN) and l-4-aminoarabinose (l-Ara4N) can also be observed. (B) Cylindrical and conical lipid A molecular shapes can be assumed by various lipid A subtypes, and these are predicted to adopt either lamellar (L)- or inverted type II hexagonal (H_II_)-phase structures, respectively. A balanced mixture of these two types of lipid A structures helps to determine the aggregate bilayer architecture of the outer membrane. Cubic phases (not shown) are also possible. Magenta circles represent glucosamine units, small red circles represent phosphate moieties, the blue diamond represents l-Ara4N, the green oval represents pEtN, and black wavy lines represent acyl chains.

In their recent report, Bonnington and Kuehn (1) build on prior work from the laboratory of Christian Raetz (2), their late colleague from the same department, who elucidated the Raetz pathway for lipid A biosynthesis (3). Focusing on *Salmonella enterica* serovar Typhimurium grown under conditions that either induce or repress the PhoPQ and PmrAB signaling pathways, Bonnington and Kuehn observed the same lipid A modifications reported earlier by Gibbons et al. in the Raetz lab (2), namely, lipid A species bearing phosphoethanolamine (pEtN) and l-4-aminoarabinose (l-Ara4N) moieties attached to the phosphate substituents and the *S*-2-hydroxylation (*S*-2-OH) of myristate and incorporation of palmitate (C_16_) in the acyl chain domain (Fig. 1A). By adopting this previously established model system, Bonnington and Kuehn spared themselves from having to repeat the biochemistry that had already been done for them. The authors’ unique contribution lies in their efforts to quantify the distribution of the various lipid A molecular subtypes located within the outer membrane and the outer membrane vesicle (OMV) fractions. The inherent complexity of the *S*. Typhimurium system makes it challenging work, but the best efforts of these investigators have now established that lipid A species bearing the pEtN and l-Ara4N moieties tend to be maintained in the outer membranes but that the lipid A species bearing C_16_ are more likely to be found in the OMV fraction. If the modified lipid A species had no influence on OMV formation, then a random distribution in the two fractions was anticipated. The most obvious explanation for why this nonrandom distribution of lipid A is observed comes from the established model for polymorphic regulation of membrane lipid composition (4, 5), which has accounted for membrane glycerophospholipid composition (6) but has yet to be adapted for considering lipid A and its modified forms present in bacterial outer membranes.

The pioneering lipid structural studies of Luzzati and colleagues (7) established that biological membranes include a mixture of both bilayer-forming or lamellar (L)-phase lipids and nonbilayer lipids, most commonly inverted type II hexagonal (H_II_)-phase lipids. The polymorphic concept states that there is a direct relationship between the cross-sectional molecular shape of any particular lipid species and the macroscopic structure of its corresponding lipid aggregate. Most simply, lipids with a cylindrical shape tend to adopt lamellar structures, whereas lipids with a conical shape (with the lipid head group on the small end of the cone) tend to adopt hexagonal structures (Fig. 1B). Brandenburg and coworkers have shown that lipid A molecules that adopt cylindrical shapes tend to be inactive as endotoxins but that those that are the most endotoxic tend to be more conical and tilted in shape (8, 9). The crystal structures of the TLR4–MD-2 complexes with various lipid A analogues reveal the molecular bases for how lipid A structure influences the innate immune response (10, 11), but lipid A evolved to function in the outer membranes of Gram-negative bacteria long before the evolution of multicellular eukaryotes. Ernst Rietschel reminded his audience of this point in his opening address to the 14th Biennial Meeting of the International Endotoxin and Innate Immunity Society in Hamburg, Germany, on 22 September 2016. What, then, are the various lipid A structures doing for the outer membranes of Gram-negative bacteria?

All biological membranes undergo essential processes where the lipid bilayer must be transiently disrupted; the formation of OMVs provides a case in point because a phase transition is expected to occur as vesicles bud from the outer membrane. To be certain how lipid A modifications influence the phase behavior of outer membranes, molecular structures of lipid A, with and without its various modifications, need to be solved in order to calculate physical constants, like the critical packing parameter used to predict L and H_II_ behavior (4, 12). Critical packing parameters are known for most of the glycerophospholipids, but until recently, only the structures of the unmodified hexa-acylated lipid A and its precursor from enterobacteria, lipid IV_A_, had been cocrystallized in certain membrane protein structures. A recent porin structure from the group of Jeremy Lakey has now revealed a hepta-acylated lipid A partial structure with C_16_ esterified to the proximal glucosamine unit (13). As expected, the hepta-acylated lipid A has a larger cross-sectional molecular shape than its hexa-acylated counterpart, and it takes on a perceptible increase in conical shape. In accordance with the predictions of Brandenburg and coworkers (8, 9), we expect palmitoylated lipid A to stimulate an increase in the inflammatory response. Indeed, when C_16_ is esterified to the distal glucosamine unit in *Pseudomonas aeruginosa* lipid A, an increase in the inflammatory response is observed, and this is thought to result from a superior ability of this modified lipid A to bind within the MD-2 receptor of the TLR4–MD-2 complex (14). However, C_16_ in enterobacterial lipid A is positioned where it will be draped over the exterior of its MD-2 receptor and block the subsequent dimerization of TLR4, thus reducing the inflammatory response in this particular case (11). An apparent exception to the rule of lipid A polymorphism for explaining signal transduction is thereby understood in molecular terms. Can lipid A polymorphism also provide a means for explaining bacterial membrane architecture? Structures of lipid A with modified phosphate groups are not yet known, but they will likely increase the volume of the head group and adjust their L or H_II_ propensity in accordance with their degree of acylation. Even without critical packing parameters calculated for the various lipid A modifications, we can still make educated guesses as to how they might behave.

A case in point concerns the role of divalent cations in controlling outer membrane stability. This is an important point to consider in the study of Bonnington and Kuehn because they were adjusting Mg^2+^ between micromolar and millimolar concentrations to switch the PhoPQ system on and off, respectively. It is well known that cardiolipin and phosphatidic acid undergo an L-to-H_II_ transition upon addition of divalent cations (12). The negatively charged polar head groups of these anionic glycerophospholipids have their surface areas reduced by roughly one-half when their charges are neutralized upon divalent cation binding. The finding that *Escherichia coli* mutants deficient in the synthesis of their major H_II_ lipid phosphatidylethanolamine respond with a large compensatory increase in anionic glycerophospholipids but become absolutely dependent on millimolar divalent cation concentrations in the growth medium remains one of the best case studies in support of the polymorphic regulation of membrane phospholipid composition (15, 16). *E. coli* also increases the incorporation of C_16_ into its lipid A after EDTA treatment of growing cells (17), and this might logically be interpreted as an expansion of the volume of the lipid A acyl chain domain in order to compensate for a corresponding increase in the volume of the lipid A polar phosphate moieties once they have been stripped of their neutralizing divalent cations. Similar explanations can likely be extended to *S*. Typhimurium, where multiple molecular subtypes of lipid A coexist in the same membrane under conditions of PhoPQ activation.

One lipid A modification not observed by Bonnington and Kuehn (1) results from the activity of the latent lipid A 3-*O* deacylase PagL. Mario Feldman’s group has recently reported that deregulated expression of PagL stimulates the formation of OMVs enriched in 3-*O*-deacylated lipid A (18). 3-*O*-Deacylation should reduce the volume of the lipid A acyl chain domain, but it also introduces a hydroxyl group, which can donate a hydrogen bond to strengthen lateral lipid interactions, as is thought to be the case following *S*-2-hydroxylation of myristate (19). Importantly, these two reports now independently confirm the potential role for polymorphic regulation of outer membrane lipid A composition in the formation of OMVs. We can all appreciate that bacteria induce lipid A modifications to modulate the host’s innate immune response to infections, but a likely consequence is that the integrity of their outer membranes is also affected (20), and one compensatory mechanism clearly involves the release of OMVs.
